# Impact of cognitive decline on the physical functioning of older adults in Southern Brazil: a cross-sectional study

**DOI:** 10.3389/fnagi.2025.1549764

**Published:** 2025-06-18

**Authors:** Andréa Kruger Gonçalves, Valéria Feijó Martins, Ana Carolina Kanitz, Flávia Gomes Martinez, Leonardo Alexandre Peyré-Tartaruga, Raphael Machado Castilhos, Aline Nogueira Haas

**Affiliations:** ^1^Centro de Referência do Envelhecimento e Movimento (CREM), Universidade Federal do Rio Grande do Sul, Porto Alegre, Brazil; ^2^Department of Public Health, Experimental and Forensic Medicine, University of Pavia, Pavia, Italy; ^3^Neurology Service, Hospital de Clínicas de Porto Alegre, Porto Alegre, Brazil; ^4^Global Brain Health Institute, Trinity College Dublin, Dublin, Ireland

**Keywords:** physical function, cognitive status, aging, dementia, physical activity

## Abstract

**Introduction:**

Reduced physical function has been linked to cognitive decline, yet this relationship remains understudied in the older Brazilian population. This study evaluates the impact of cognitive decline on physical functioning in older adults in Southern Brazil.

**Methods:**

A cross-sectional analysis was conducted with community-dwelling older adults (*n* = 336), categorized as cognitively unimpaired (CU, *n* = 181), mild cognitive impairment range (MCI-range, *n* = 105), or dementia-range (*n* = 50) based on Montreal Cognitive Assessment cutoff points adapted for the Brazilian population. Physical function was assessed using tests for balance, strength, flexibility, and aerobic endurance. Statistical analyses included one-way ANOVA with *post hoc* Bonferroni tests.

**Results:**

Most physical function variables worsened as cognitive impairment increased. The CU group performed significantly better than the MCI- and dementia-range groups in dynamic and static balance, upper-body strength (*η^2^* = 0.09), upper- and lower-body flexibility (η^2^ = 0.07, *η^2^* = 0.08), and aerobic endurance (*η^2^* = 0.11) (*p* < 0.05).

**Conclusion:**

Cognitive status influences physical function in older adults. Identifying physical indicators linked to cognitive decline may improve screening efforts in clinical trials.

## 1 Introduction

The World Health Organization (WHO) estimates that by 2050, the global population of individuals aged 60 and older will reach nearly 2 billion (United Nations Population Division, 2019), a shift closely linked to a rise in mild cognitive impairment (MCI-range) and dementia-range (Chen et al., 2024). In Brazil, it is projected that 6 million people will be living with dementia by 2050 (Nichols et al., 2022). This growing burden presents a significant challenge to healthcare systems and societies worldwide (Duran-Aniotz et al., 2022). Moreover, low- and middle-income countries in Latin America, including Brazil, face a higher prevalence of cognitive decline compared to high-income countries, compounding their socioeconomic and health care challenges (Ribeiro et al., 2021; Suemoto et al., 2023).

Cognitive function exists on a continuum, typically classified into three categories: cognitively unimpaired (CU), mild cognitive impairment (MCI-range), and dementia-range (Cesar et al., 2016; Dautzenberg et al., 2022). CU refers to preserved cognitive function with no signs of impairment. MCI-range represents an intermediate stage, where individuals experience cognitive decline (e.g., memory deficits) but maintain independence (Gauthier et al., 2006; Petersen, 2011). Dementia involves more severe cognitive impairment, leading to loss of independence and the capacity to perform activities of daily living (Gauthier et al., 2006; Petersen, 2011).

Reduced physical function is closely tied to cognitive decline (Chen, 2023; Mielke et al., 2013). Slower gait speed is associated with cognitive decline and dementia, suggesting it may serve as an early indicator of cognitive risk, as gait relies on motor, perceptual, and cognitive functions (Mielke et al., 2013). Impairments in balance, coordination, and lower body functionality are linked to age-related alterations in the central nervous system, affecting sensorimotor processing and integration (MacDonald et al., 2017; Montero-Odasso et al., 2014). Motor task changes, including strength, gait, and balance, consistently correlate with cognitive impairment and the potential for dementia (Guerrero-Berroa et al., 2014). For instance, muscle strength may reflect central nervous system integrity and aging-related changes (Kim et al., 2024), and cardiorespiratory fitness shows associations with cognitive status, with higher fitness levels linked to better cognitive function and a reduced risk of cognitive decline (Sobol et al., 2018). These diverse factors collectively emphasize the intricate link between physical function and cognitive status, offering potential insights for intervention in cognitive decline and aging.

Considering the above, there is a gap in understanding the relationship between the different components of physical function and cognitive status within the older in Brazilian population. In international studies, the results are inconclusive and do not distinguish between different cognitive status (Handing et al., 2020; Liu et al., 2021; Zhao et al., 2022). To the author’s knowledge, no studies have considered Brazilian older adults’ physical function and different cognitive status.

Studying older adults with cognitive decline across different regions of Brazil is essential due to the country’s unique socio-economic factors, such as disparities in healthcare access and educational background, which may influence aging process (Ribeiro et al., 2021; Suemoto et al., 2023). Research in this context enhances the global understanding of aging and cognitive health, particularly in settings beyond high-income countries, contributing to the development of culturally and contextually appropriate interventions. Therefore, this study aims to evaluate the impact of cognitive status on the physical functioning of older adults in Southern Brazil. The Southern Brazilian region faces structural challenges common to many Latin America countries and to other Brazilian regions, such as inequalities and an aging population. The scarcity of region-specific data is identified as a key rationale for including this population in the study.

## 2 Materials and methods

### 2.1 Study design and participants

The present study is an observational study, with a cross-sectional analysis, in which baseline data was extracted from a controlled, randomized, and interdisciplinary clinical trial database (NCT06638697) from the Reference Center for Aging and Movement (CREM), a program at the School of Physical Education, Physiotherapy, and Dance, Federal University of Rio Grande do Sul (UFRGS), Brazil. The CREM aims to be a reference in the study of movement and aging, with the objective of evaluating the effects of different physical exercise modalities, such as gymnastics, hydro gymnastics, strength training, dance, Nordic walking, and aquatic jogging, for older adults and developing research about its effects on clinical and functional outcomes in older adults. This study was approved by the local research ethics committee (Protocol ID 65435022.9.0000.5347). Participants from the CREM/UFRGS voluntarily enrolled in the study and received comprehensive information regarding the research objectives and methods. All the participants provided informed consent before participation.

The study’s eligibility criteria included all individuals aged 60 years or older, of both sexes, capable of understanding verbal instructions, and recruited from the CREM/UFRGS, forming a convenience sample. Exclusion criteria were as follows: not engaging in regular physical exercise for at least 3 months prior to the start of the research, and not having medical conditions contraindicating participation, such as severe heart disease, uncontrolled diabetes type I and hypertension, recent myocardial infarction, history of physical-cognitive impairment or stroke sequelae, presence of a pacemaker, recent surgery, lower body prostheses, or neuropathic pain in the lower body.

### 2.2 Procedure

The participants were invited to join based on an in-person meeting held at the beginning of the CREM/UFRGS activities. Every participant completed all assessments in 1 day, conducted by trained researchers specialized in administering the tests. Sociodemographic data were collected, including age (years), sex, race (self-identified), socioeconomic status (self-reported family income in relation to the country’s minimum wage), education level (school qualification), and marital status to characterize the participants.

#### 2.2.1 Cognition

Global cognition was assessed using the Montreal Cognitive Assessment (MoCA), a widely used screening tool, to categorize participants based on cognitive function and identify those at risk of impairment (Nasreddine et al., 2005). The MoCA covers various cognitive domains (visuospatial/executive, naming, memory, attention, language, abstraction, delayed recall, and orientation). Scores on the MoCA can range from 0 to 30, with higher scores indicating better cognitive function. Participants were classified in cognitively unimpaired (CU), mild cognitive impairment (MCI-range), and dementia-range according to the total MoCA score. The definition of CU, MCI-range and dementia-range involves a more extensive evaluation and that a single screening test is not sufficient to define these diagnoses. However, these nomenclatures were maintained to facilitate understanding.

The cut-off points of the MoCA were adjusted according to education (Cesar et al., 2019): for those with ≥ 12 years of education (CU group ≥ 24 points; MCI-range group 23– 21 points; dementia-range group ≤ 20 points); from 9 to 11 years (CU group ≥ 23 points; MCI-range group 22–13 points; dementia-range group ≤ 12 points); from 5 to 8 years (CU group ≥ 21 points; MCI-range group 20 to 13 points; dementia-range group ≤ 12 points); and from 1 to 4 years (CU group ≥ 18 points; MCI-range group 17– 12 points; dementia-range group ≤ 11 points). Adapting cognitive screening to individuals’ education levels is vital, as education directly impacts cognitive abilities (Feldberg et al., 2024). Older adults with diverse levels of education in Brazil showed significant variations in the Montreal Cognitive Assessment (MoCA) scores according to age and education (Cesar et al., 2019). Additionally, cutoff points for the Brazilian population differentiating cognitive impairment from no cognitive impairment were established according to level of education (Cesar et al., 2019).

#### 2.2.2 Physical function

A series of physical function variables assessments were performed, including tests for dynamic and static balance, upper- and lower-body strength, upper- and lower-body flexibility, and aerobic endurance (Rikli and Jones, 2013). These tests included the Balance and Agility Test, single-leg stance test, arm-curl test, 30-sec chair-stand test, back-scratch test, chair sit-and-reach test, and the 6-min walk test, providing a comprehensive evaluation of the participants’ physical function.

The Balance and Agility Test proposed by Rikli and Jones (2013) assessed dynamic balance. The subject stands up, walks 2.44 m, turns around, and returns, finishing the test by sitting down again. The performance was measured in seconds (sec) for the time to complete the task. Static balance was assessed through the single-leg stance test. The test evaluated the individual’s ability to maintain a unipedal stance with eyes open and subsequently with eyes closed. The performance with eyes open and closed was measured by the time, in seconds (sec), maintained in the balance position, with a maximum time of 30 sec.

Upper-body strength was assessed through the arm-curl test. The participant assumed a seated posture with an upright back and feet resting on the floor and flexed and extended the elbow holding 2 kg for women and 4 kg for men for 30 sec. The total repetitions completed are recorded (Rikli and Jones, 2013). The 30-sec chair-stand test to assess lower body strength was used. The participants started sitting in a chair, feet on the floor, and arms crossed over the chest. The participants rose from and returned to the chair as many times as possible within 30 sec. The performance of both tests was measured by the total number of repetitions (rep) completed in 30 sec (Rikli and Jones, 2013).

Upper-body flexibility was assessed through the back scratch test. While standing, the participant placed their preferred hand over the same shoulder, with an open palm and extended fingers, reaching as far as possible toward the middle of the back. The hand of the other arm was positioned behind the back, palm facing upward, attempting to reach as far as possible to touch or overlap the extended middle fingers of the opposite hand. The chair sit-and-reach test was used to assess lower-body flexibility in which the participants remained seated on a chair with one leg extended and attempted to touch their toes. The performance of both tests was measured in centimeters (cm), based on the distance reached during the stretch (Rikli and Jones, 2013).

Aerobic endurance was assessed through the 6-min walk test. A 30-m course is marked out, and the participants were instructed to walk as swiftly as possible (without running) within the allotted time. The performance was measured in meters (m), based on the total distance covered during the 6-min walking period (Rikli and Jones, 2013).

### 2.3 Data analysis

The participants’ demographic characteristics were descriptively analyzed. A Shapiro-Wilk test was conducted to assess the data normality. The ANOVA one-way was used to determine the main effects of the different cognitive status groups (CU, MCI-range and dementia-range). A Bonferroni correction for multiple comparisons was used for the comparisons between groups. Additionally, unadjusted and adjusted logistic regression models were conducted to examine the contribution of physical function and demographic characteristics to cognitive decline. The unadjusted model included only physical function variables, while the adjusted model incorporated demographic variables. For the linear regression model, the continuous MoCA score was used as the primary outcome, and the independent variables were included according to their respective units of measurement (e.g., dynamic balance was expressed in sec). All statistical analyses were performed using JASP for macOS version 0.17.1 (University of Amsterdam).^1^

## 3 Results

### 3.1 Participants characteristics

Three hundred and thirty-six participants (270 females and 66 males) from the CREM/UFRGS who were eligible for the study were included in the analysis. Participants were divided into three groups according to the MoCA and its cutoff points, adjusting according to education and adapting to the Brazilian population (Cesar et al., 2019): CU (181; 54%), MCI-range (105; 31%), and dementia-range (50; 15%).

The participants’ demographic characteristics are shown in Table 1. Most of the participants were females, white, married, with ≥ 12 years of education, and income between 1 and 3 minimum wage, except for the dementia-range group which was 4–6 minimum wage (MW).

**TABLE 1:** Participants’ sociodemographic characteristics.

		CU (*n* = 181)	MCI-range (*n* = 105)	Dementia-range (*n* = 50)
Age (years) (mean ± SD)		70.4 ± 6.4	74.5 ± 7.4	72.8 ± 6.5
**MoCA (total score) (mean ± SD)**		24.8 ± 2.2	19.7 ± 2.8	17.5 ± 3.3
Sex n (%)	Female	150(82.8)	86(81.9)	34(68.0)
	Male	31(17.2)	19(18.1)	16(32.0)
Race* n (%)	White	151(83.4)	71(67.6)	36(72.0)
	Black	9(4.9)	11(10.4)	8(16)
	Mixed race	4(2.2)	9(8.5)	–
Marital status* n (%)	Married	75(41.4)	33(31.4)	26(52.0)
	Single	24(13.2)	16(15.2)	3(6.0)
	Divorced/separated	35(19.3)	14(13.3)	10(20.0)
	Widower	30(16.5)	28(26.6)	5(10.0)
Education* n (%)	1–3 years	3(1.6)	2(1.9)	–
	4–7 years	13(7.1)	17(16.1)	3(6.0)
	8–11 years	38(20.9)	30(28.5)	1(2.0)
	≥ 12 years	127(70.1)	56(53.3)	46(92.0)
Income* n (%)	≤ 1 MW	20(11.0)	13(12.3)	4(8.0)
	1–3 MW	56(30.9)	36(34.2)	12(24.0)
	4–6 MW	43(23.7)	21(20.0)	13(26.0)
	≥ 7 MW	19(10.5)	8(7.6)	7(14.0)

*Presents missing or date not reported; MW: minimum wage, minimum wage approximately $250.

### 3.2 Physical function in different cognitive status

The main factors showed that, statistical difference between the different cognitive status groups (Table 2) was found in the dynamic balance (*F* = 6.927; *p* = 0.001); static balance eyes open (*F* = 3.977; *p* = 0.020); static balance eyes closed (*F* = 4.876; *p* = 0.008); upper-body strength (*F* = 5.428; *p* = 0.005); upper-body flexibility (*F* = 9.688; *p* < 0.001); lower-body flexibility (*F* = 6.435; *p* = 0.002); aerobic endurance (*F* = 4.949; *p* = 0.008). The majority of the physical function variables were deteriorated with increasing cognitive impairment. The CU group was faster in performing dynamic balance compared to the MCI-range group (*p* = 0.003) and dementia-range (*p* = 0.043). The CU group spent more time in single-leg stance static balance with eyes open than the MCI-range group (*p* = 0.019), the same happened for static balance with eyes closed (*p* = 0.014). In the upper-body strength test, the CU group was stronger compared to MCI-range (*p* = 0.007), with the same behavior in the upper-body flexibility (*p* < 0.001). In the lower-body flexibility, the CU group was more flexible than the dementia group (*p* = 0.003). In aerobic endurance, the CU group walked a greater distance than the MCI-range group (*p* = 0.017).

**TABLE 2:** Mean values of the aspects physical in older adults.

Variables	CU (*n* = 181)	MCI-range (*n* = 105)	Dementia-range (*n* = 50)	*p*-value
Dynamic balance (sec)	5.5 ± 1.8	6.3 ± 2.1	6.3 ± 1.8	0.001*
Static balance eyes open (sec)	18.0 ± 11.1	14.2 ± 11.0	15.5 ± 11.4	0.020*
Static balance eyes closed (sec)	4.0 ± 3.7	2.8 ± 2.6	2.9 ± 1.8	0.008*
Upper-body strength (rep)	21.3 ± 4.7	19.5 ± 5.1	19.7 ± 4.5	0.005*
Lower-body strength (rep)	16.4 ± 4.4	15.3 ± 4.2	15.1 ± 5.4	0.064
Upper-body flexibility (cm)	−5.2 ± 10.9	−12.0 ± 14.8	−7.2 ± 12.1	< 0.001*
Lower-body flexibility (cm)	1.0 ± 11.0	−2.1 ± 11.2	−5.2 ± 14.4	0.002*
Aerobic endurance (m)	476.0 ± 88.8	443.3 ± 103.2	442.3 ± 87.1	0.008*

sec: seconds; rep: repetitions; cm: centimeters; m: meters; *p-values refer to ANOVA (*p* < 0.05) indicate significant difference.

**FIGURE 1:**
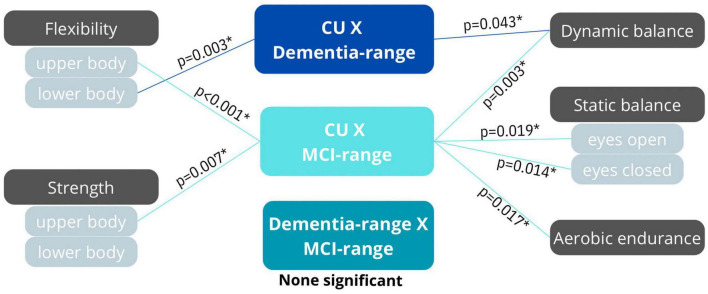
Mean of the physical functional variables, results of the test of main effects by ANOVA one-way and pairwise comparisons by Bonferroni’s *post-hoc* test. Groups cognitively unimpaired (CU), mild cognitive impairment (MCI-range), and dementia-range; Horizontal black lines represent statistical differences between groups; The symbol * represents the general difference between groups in ANOVA.

Figure 1 shows the participants’ physical function in the different cognitive status and the results of the adjusted analysis.

**FIGURE 2:**
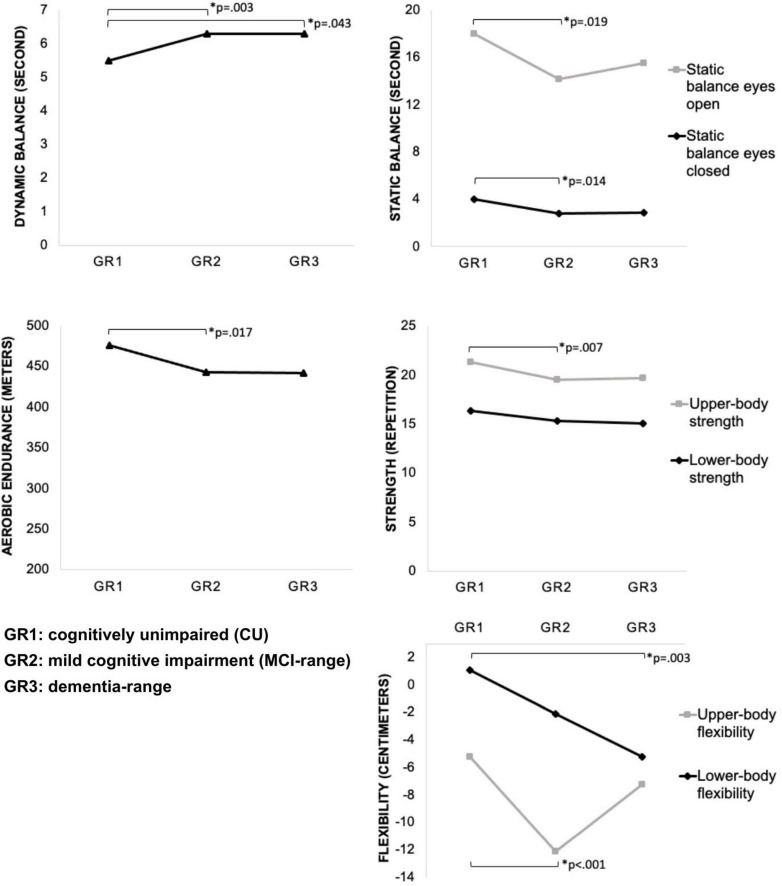
Representation of the between groups results. *Indicate significant difference between (*p* < 0.05).

The CU group showed differences in all the study variables compared to the other two groups: it was different from the MCI-range group in all six variables and from the dementia-range group in three variables (Figure 2). The MCI-range group showed differences exclusively with the CU group (in all variables), while the dementia-range group showed differences with the CU group only in the three mentioned variables (Figure 2).

A linear regression analysis was conducted to assess the direct effect of physical function on cognition (Table 3). The regression model was statistically significant (*F* = 9.805, *p* < 0.001) when the physical function variables were included, explaining 20% of the variance in cognition (adj. *R^2^* = 0.207), with dynamic balance (β = 0.22) and upper-body flexibility (β = 0.15) as significant predictors. Additionally, the regression model remained statistically significant (*F* = 12.149, *p* < 0.001) when the independent variables of age, sex, and education level were introduced, explaining 31% of the variance in cognition (adj. *R*^2^ = 0.310). Dynamic balance remained a significant predictor (0.16 points) when sociodemographic variables were controlled (age: −0.29 points; education level: 0.11 points). The regression analysis showed that for each additional sec in the dynamic balance test, cognition decreased (β = −0.16); for each additional year of age, cognition decreased (β = −0.29); and for each additional year of education, cognition increased (β = 0.11).

**TABLE 3:** Physical function and demographic characteristics (age, sex, and education level) to explain cognition.

Independent variables	Regression step 1		Regression step 2	
	β	*p*	β	*p*
Dynamic balance	−0.223	< 0.001*	−0.168	0.024*
Static balance eyes open	0.086	0.155	0.050	0.383
Static balance eyes closed	0.015	0.786	0.016	0.762
Upper-body strength	0.003	0.964	0.015	0.800
Lower-body strength	−0.025	0.696	−0.002	0.973
Upper-body flexibility	0.157	0.013*	0.074	0.229
Lower-body flexibility	0.017	0.764	0.042	0.461
Aerobic endurance	0.117	0.101	0.070	0.303
Age	–	–	0.291	< 0.001*
Sex (men)	–	–		0.248
Education level	–	–	−0.113	0.041*
F	9.805 (*p* < 0.001)		12.149 (*p* < 0.001)	
r2	0.455		0.557	
Adj r2	0.207		0.310	

*p-values (*p* < 0.05) indicate significant difference.

## 4 Discussion

The purpose of this study was to evaluate the impact of cognitive status on the physical functioning of older adults in Southern Brazil. The results showed that physical function differed between the groups, with CU participants outperforming those with dementia-range, confirming our hypothesis and previous findings. Participants with CU exhibited better dynamic balance, static balance, upper-body strength, upper-body flexibility, lower-body flexibility, and aerobic endurance compared to those with MCI-range and dementia-range. Additionally, the regression analysis showed that dynamic balance and upper-body flexibility were significant predictors of cognitive function, explaining 20% of the variance. After adjusting for age, sex, and education level, dynamic balance remained a significant predictor. These findings suggest that cognitive status is associated with physical function among older adults. Another important finding was the lack of differences between the MCI-range and dementia-range groups, suggesting that further investigation is needed to uncover the nuances of cognitive impairment in this context.

The observed decline in physical function with increasing cognitive impairment aligns with existing literature, emphasizing the interconnectedness of cognitive and physical health in aging populations (Chen, 2023; MacDonald et al., 2017; Mielke et al., 2013). Neurodegenerative processes, such as those associated with dementia-range, can impair motor control and coordination, leading to gait disturbances and an increased risk of falls (Bahureksa et al., 2016; Pitts et al., 2023). Additionally, cognitive decline may reduce motivation and engagement in physical activities, as cognitive changes can impact one’s ability to plan, make decisions, and remember, which are key factors for participating in such activities (Fallahpour et al., 2016). While physical activity is associated with the prevention of cognitive decline, a decrease in cognitive function may hinder adherence and participation, creating a vicious cycle in which reduced engagement in physical activities further exacerbates cognitive decline (Fallahpour et al., 2016). Furthermore, studies have consistently shown that cognitive decline is often accompanied by impairments in motor function and balance (Guerrero-Berroa et al., 2014; Montero-Odasso et al., 2014). In older adults, gait speed, balance, and body strength are associated with MCI-range in the Chinese population (Liu et al., 2021). Similarly, in the Japanese population, lower body strength also shows this association (Iwamoto et al., 2024). Another study found similar results regarding strength and aerobic endurance (Zhao et al., 2022), but no association was observed with mobility, flexibility, and balance. In Brazil, no studies were found for comparison.

As noted in the introduction, few studies evaluated older adults with different cognitive status, underscoring the early stage of research in this area. Thus, the mechanisms underlying the relationship between physical and cognitive function remain unclear. The association between physical and cognitive function are complex and multifaceted, involving both biological and psychosocial factors. A recent study linked physical exercise with physical, emotional, and cognitive health in older adults over an 8-year period showed inconsistent results regarding cognition, as the sensitivity analysis suggests that the positive association between regular physical exercise and cognitive function may not be sufficiently valid (Xu et al., 2024).

The relationship between physical and cognitive functioning differs in its components and domains, suggesting more specific rather than general relationships (Liu et al., 2021). Growth factors such as insulin-like growth factor 1, brain-derived neurotrophic factor, and lower inflammation are influenced by physical activity (Zhao et al., 2022). Conversely, the progression of dementia significantly impairs functional fitness, affecting various aspects of physical performance (Jiménez-García et al., 2024; Makizako et al., 2022; Xu et al., 2024; Zhao et al., 2022). As dementia advances, there is a marked decline in the ability to perform activities of daily living due to the deterioration of both cognitive and physical functions (Sommerlad and Rapaport, 2021). Cognitive impairment, particularly in attention and processing speed, have been strongly correlated with decreased physical performance, impacting the execution of daily activities (Clemmensen et al., 2020). Our results suggest a similar relationship between physical performance and cognitive function, which may also be linked to the performance of daily activities. Although this was not the primary focus, our intention was to highlight additional perspectives beyond just physical function.

In most of the variables in our study, significant differences were observed between the CU group and those with some level of impairment (MCI-range and dementia-range). The execution of physical tasks, such as those evaluated in this study, requires a complex interaction of various systems (including the musculoskeletal, sensory, and cognitive systems), which may be impaired in individuals with cognitive impairment (Montero-Odasso et al., 2014; Xu et al., 2024; Zhao et al., 2022). Thus, physical fitness results can serve as indicators of whether an older adult may have cognitive impairment (Jiménez-García et al., 2024; Liu et al., 2021; Zhao et al., 2022). Consequently, motor planning and execution, often compromised in these individuals, can lead to reduced physical performance, an increased risk of accidents, and a decline in overall functional independence (Ali et al., 2022; Pitts et al., 2023).

Physical function was assessed based on six components. Among the possible group comparisons (CU vs. MCI-range, CU vs. dementia-range, MCI-range vs. dementia-range), the first two revealed differences in two components: dynamic balance and flexibility. Cognitive impairment affects the brain regions responsible for planning and executing complex movements, which can result in difficulties with balance, walking, and performing dual tasks. Cognitive-motor interference is more pronounced in individuals with cognitive impairment, leading to greater challenges in maintaining balance and gait stability (Pitts et al., 2023).

Muscle strength differed between the CU and MCI-range groups. Cognitive impairment can impact muscle strength by disrupting the brain’s ability to send accurate signals to the muscles, leading to reduced activation, coordination, and control, increasing the risk of falls and injuries (McGrath et al., 2019; Zha et al., 2022). Strength is associated with cognitive status due to the complex cognitive functions involved in muscle activation and coordination (Sommerlad and Rapaport, 2021).

The CU group exhibited better aerobic endurance compared to the MCI-range group. Higher endurance is linked to increased cerebral blood flow, which supports brain cell oxygenation and nourishment (Martins et al., 2024; Zhao et al., 2022), suggesting better cognitive function and a more effective brain network (Kawagoe et al., 2017).

The CU group also showed greater flexibility in both the upper and lower body compared to the MCI-range and dementia-range groups. Flexibility relies on the central nervous system’s ability to coordinate muscle relaxation and contraction, and cognitive decline can impair this coordination. This can result in limited movement, affecting activities involving bending, stretching, or reaching (McGrath et al., 2019). Although there is a scarcity of studies addressing these variables, existing research indicates that cognitive impairment may contribute to decreased flexibility, with inconclusive findings in some areas (Matos et al., 2020).

Finally, the CU group demonstrated superior dynamic and static balance compared to those with MCI-range and cognitive impairment. Notably, dynamic balance was a significant predictor of cognitive function, highlighting the strong connection between the two. Previous studies show that cognitive decline negatively impacts balance (Qi et al., 2023; Song et al., 2023), with proprioception and sensory input, essential for balance, deteriorating alongside cognitive impairment. Our results align with these observations, indicating that better cognitive function is associated with improved balance. Additionally, a systematic review supports our findings, showing a significant link between executive function and dynamic balance, emphasizing the role of cognitive processes in maintaining balance and reinforcing the connection between them (Divandari et al., 2024),

The strengths of this study include a comprehensive assessment of a highly functional older population of Southern Brazil, using validated protocols for both physical and cognitive function. The detailed physical assessment battery (Rikli and Jones, 2013) used in this study represents a key strength of the design, providing robust and reliable data for evaluating the relationship between physical function and cognitive impairment. To the authors’ knowledge, this is the first study conducted in Southern Brazil with this population, considering regional and socio-cultural factors that may influence aging and health outcomes. As such, it can serve as a valuable tool for screening older Brazilian adults at risk of cognitive impairment and contribute to comparisons with international cohorts. This is a recent and important area of research in healthcare, where these variables may be sensitive in detecting early signs of dementia, supporting targeted interventions and public health strategies.

However, some limitations need to be addressed. The sample was drawn from a specific region in Southern Brazil, with socioeconomic development indices higher than those of other regions in the country (Suemoto et al., 2023), which may not fully represent the diverse Brazilian or other Latin American countries population. Variations in socioeconomic status, healthcare access, and cultural factors across Brazil could influence both physical and cognitive health, potentially limiting the generalizability of the findings. Future studies should include diverse populations from multiple Brazilian regions and other Latin American countries, utilizing multi-site designs, stratified sampling, and adjustments for socioeconomic and cultural factors. Additionally, cross-sectional design limits the ability to establish a causal relationship between cognitive decline and physical function. To address this limitation, future longitudinal studies are necessary to observe changes in cognitive status and physical function over time, allowing for the examination of causal relationships and directional effects between the two variables.

In conclusion, this study findings showed that CU older adults exhibit better physical function across multiple domains, including balance, strength, flexibility, and aerobic endurance, compared to their cognitively impaired counterparts. While no significant differences were detected between the MCI and dementia groups, the findings suggest that physical function may serve as an important indicator of cognitive impairment. These results highlight that preserving physical health may play a crucial role in maintaining overall cognitive function in older populations. Future studies should aim to refine the methods for assessing this relationship and explore their clinical applicability, particularly in improving early detection and monitoring in populations at risk of cognitive decline.

## Data Availability

The raw data supporting the conclusions of this article will be made available by the authors, without undue reservation.

## Footnotes

1
https://jasp-stats.org/
